# Molecular and morphological characterization of *Xylaria karsticola* (*Ascomycota*) isolated from the fruiting body of *Macrolepiota procera* (*Basidiomycota*) from Bulgaria

**DOI:** 10.1371/journal.pone.0287679

**Published:** 2023-06-29

**Authors:** Galena Angelova, Petya Stefanova, Mariya Brazkova, Albert Krastanov

**Affiliations:** Department of Biotechnology, University of Food Technology, Plovdiv, Bulgaria; ICAR-Directorate of Mushroom Research, INDIA

## Abstract

The present study is the first to report *Xylaria karsticola* isolated from the basidiocarp of *Macrolepiota procera* (*Basidiomycota*), from Stara Planina Mountain, Bulgaria and second report for such species found in Europe. The fungal isolate was *in vitro* cultivated and the morphology was observed. It was primarily determined as a xylariaceous morphotype at the intragenus level, based on the evaluation of colony growth rate, color, and stromatic structure formation and was confirmed by unique conidiophores and conidia. The molecular identification of the isolate was performed by amplification of ITS1-5.8S-ITS2 region and the strain was identified as *Xylaria karsticola* with 97.57% of confidence. The obtained sequence was deposited in the GenBank database under the accession number MW996752 and in the National Bank of Industrial Microorganisms and Cell Cultures of Bulgaria under accession number NBIMCC 9097. The phylogenetic analysis of the isolate was also conducted by including 26 sequences obtained from different *Xylaria* isolates. Considering the phylogenetic data, *X*. *karsticola* NBIMCC 9097 was grouped along with other *X*. *karsticola* isolates, although the DNA sequence of the novel *X*. *karsticola* was rather distantly related to the other *X*. *karsticola* sequence data. The results were supported by the bootstrap analysis (100%) and indicated the different origin of the examined *X*. *karsticola* NBIMCC 9097.

## Introduction

*Xylaria* Hill ex Schrank is the most common and diverse genus of *Xylariaceae* (*Xylariales*, *Sordariomycetes*) family of *Ascomycota* phylum and includes about 819 accepted name records listed in *Index Fungorum* [1–5]. There are no evidences of this genus to have been monographed by polythetic methodology and because of that it is possible to comprise much more unrecognized and formally undescribed species [5]. One of the distinctive features of *Xylaria* genus is the formation of relatively large macroscopic stromata [6]. Often within the same stromata could occur either asexual or sexual reproducing cycles but at different time of the development. When conidia are observed on the mature stromata, they are usually remains of an earlier occurred asexual stage [7]. The tropical and subtropical regions of the Earth seem to be the preferred habitat for many *Xylaria* species [2, 3, 8, 9].

The most representatives of genus *Xylaria* are considered saprophytes, sometimes from slight to strongly parasites most often found in association with the stem and leaves and rarely on fruits [4]. Despite being saprophytes, *Xylaria* species are among the predominant fungal endophytes that can colonize asymptomatically wide range of hosts such as herbs and woody plants, seeds, fallen leaves and fruits or are the mutualistic symbionts in lichens (endolichens) at least in one phase of their life cycle [4, 9–18]. Also, some species could be found on dung or are associated with insects’ nests [11, 12, 19]. There are limited evidences about the spread of *Xylaria* species on fruiting bodies of mushrooms (*Basidiomycota*) [20]. In recent years some studies have been focused on these so-called fungicolous fungi for exploring the important microbial interaction between fungi and their mycohosts [21–25]. Fungicolous fungi are very large and important ecological group, associated with other fungi [23, 24]. It is indicated that some fungal cultures can promote fruiting body development while others may get their nutrition by acting as “decomposers” of mushroom fruiting bodies [21]. However, the studies on ascomycetous fungi associated with fruitifications of basidiomycetous fungi as well as their interactions are still very scarce [20, 24, 26, 27]. The first report of xylariaceous fungi appeared in 2016, where from over a hundred *Scytinopogon* sp. basidiomata specimens about fifteen species of xylariaceous fungi were isolated and identified by rDNA ITS sequencing [28]. The *Xylaria* fungi are significant part of natural ecosystems and play important ecological role due to their co-evolution with vascular plants as well as production of specific enzyme systems enabling them to decay wood causing soft-rot type of decomposition [2, 11, 29]. Due to the vast range of synthesized bioactive compounds xylariaceous endophytes could help their hosts to resist external biotic and abiotic stress factors and benefit its survival [30–37].

Although, the species of *Xylaria* genus are common, their identification is still a challenge to the mycologists. The classical technics for identification relying only on perithecium characterization as well as micro- and macroscopic features are sometimes not sufficient for the differentiation of closely related species [38]. In addition, during *in vitro* cultivation, some *Xylaria* species can reproduce only asexually that also limits proper identification depending only on colonial and anamorphic features observed [4]. The advanced molecular DNA sequence-based studies i.e. ITS-5.8S-ITS2 region, β-tubulin, α-actin, RPB2, etc., facilitate the species differentiation of genus *Xylaria*. [1, 4, 12]. According to Cañón et al. [4] and Senanayake et al. [18] the accuracy of identification also strongly depends on the proper field observation and providing connection between teleomorph found in nature and anamorph obtained *in vitro* culture.

The information about diversity of *Xylaria* species in the temperate regions of the world, particularly in Europe remains scarce. The most widespread and well recognized *Xylaria* species in Europe are *X*. *hypoxylon* (L.Fr.) Grev., *X*. *polymorpha* (Pers.: Fr.) Grev, *X*. *carpophila* (Pers.: Fr.) Fr and *X*. *longipes* Nitschke [39, 40]. In 2010 three new *Xylaria* species (*Xylaria karsticola*, *Xylaria vasconica* and *Xylaria cinerea* were described from southwestern Europe [2]. Rönsch et al. (2010) reported about two new *X*. *delitschii* and *X*.*oxyacanthae* found in Germany. *Xylaria violaceorosea* sp. nov [41] and *Xylaria xylarioides* [42] were discovered for the first time in Asturias and Catalonia & Galicia in Spain, respectively and *X*. *melitensis* is found in Malta [43]. There is only one record for *X*. *karsticola* in *Index Fungorum*. Our survey of literature showed no published information about isolation, morphological characterization and molecular identification of any *Xylaria* species from Bulgaria.

In the present studies, a fungal isolate from the fruiting body of *Macrolepiota procera* (*Basidiomycota*) collected in Bulgaria is subjected to morphological and cultural characterization by cultural-dependent approach and molecular identification.

## Materials and methods

### Fungal isolation, in vitro cultivation and morphological characterization

**Fig 1:**
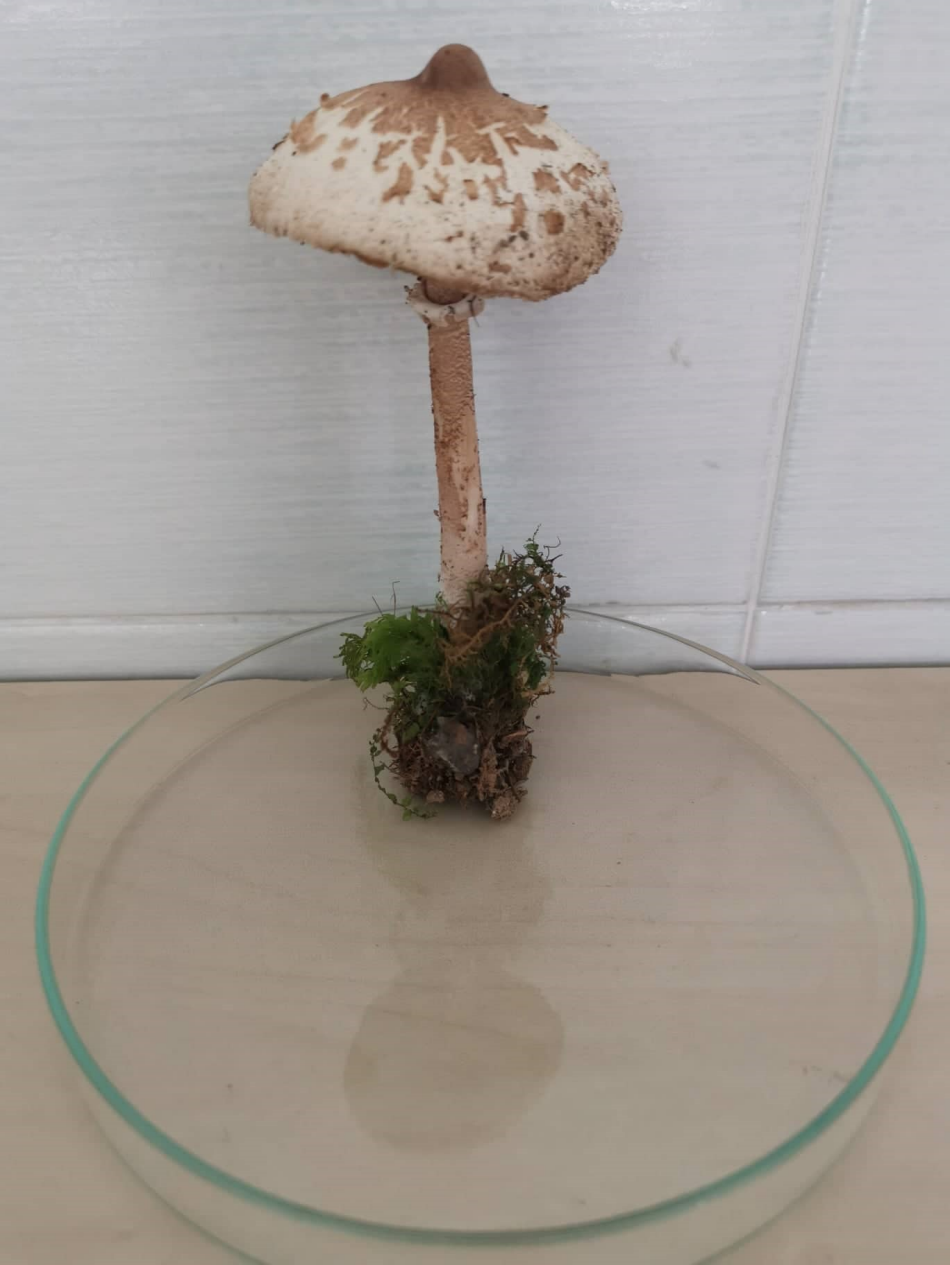
*Macrolepiota procera* (parasol mushroom).

Specimens of *Macrolepiota procera* fruiting body (Fig 1), commonly known as parasol mushroom were collected from Stara Planina Mountain, near Troyan, Bulgaria (42° 51’ 0.252" N, 24° 38’ 35.091" E) in May, 2019. Since the parasol mushroom is not included in the Red Data Book of Republic of Bulgaria and the location is not a private property no permits for the sample’s collections were required. The pilei caps from freshly collected specimens, without visible symptoms of fungal colonization, were first rinsed with tap water followed by sterile distilled water and cut into 20 to 30 mm pieces. These pieces were surface sterilized with 70% ethanol with 2–3 drops of Tween 80 for 20 sec, followed by 10 sec treatment in 2% NaClO and rinsing again in sterile distilled water. The samples were further sliced to 5 × 5 mm pieces with sterile scalpel and aseptically transferred on Rose Bengal Chloramphenicol Agar (RBCA) (HIMEDIA, India). The plates were incubated in darkness at 25°C for 14 days and were visually monitored on daily basis. The resulting unknown fungal colonies were isolated and purified by several transfers of growing mycelium on fresh medium. The unknown pure fungal isolate was maintained at 4°C on Mushroom Complete medium (MCM), containing g/L: glucose—20.0, KH_2_PO_4_−0.5, K_2_HPO_4_−1.0, MgSO_4_−0.5, peptone—2.0, yeast extract—2.0, Agar—2.0, pH 4.8–5.2 and used for further phenotypic characterization and molecular identification.

The texture and size, together with the color of the top of the colony and the bottom side of the plate and stromata produced by colonies were daily observed for 2 months. The data obtained from the morphological analysis of the stromata was documented and used together with the data of the molecular analysis for confirmation of the isolate as *Xylaria* sp. Microscopic characteristics of stromata and anamorph observation were taken using biological microscope Olympus CX43 (Olympus, Japan) after the cross-sections of the stromata were made by hand and were mounted in water or fixed with the scotch tape imprint method and stained with methylene blue.

### DNA extraction, PCR amplification, purification and sequencing

Prior to DNA extraction, the fungal isolate was cultivated for 7 days on MCM agar plates. The fungal mycelium was scraped out with a sterile spatula (100–300 mg) and transferred to a 2 ml microtube. Total DNA was extracted using a modified CTAB method, according to Stefanova et al. [44]. The quality and concentration of DNA extracts were determined by spectrophotometric measurements using Shimadzu UV-VIS spectrophotometer (Shimadzu Corporation, Japan).

The ITS-5.8S-ITS2 region was amplified by forward primer ITS 4 (5’-TCCTCCGCTTATTGATATGC-3’) and reverse primer ITS 5 (5’-GGAAGTAAAAGTGCTAACAAGG-3’) [45] obtained from Metabion (Martinsried, Germany). The PCR analysis was performed in final reaction volume of 20 μl containing 1 μl of DNA (50 ng), 0.5 μM of each primer and 8 μl of Red-Taq DNA Polymerase Master Mix (Canvax Biotech, S.L., Spain). The parameters of amplification were as follows: initial denaturation at 95°C for 10 min, 35 cycles of 1 min at 95°C, 1 min at 52°C and 1 min at 72°C, and final extension at 72°C for 7 min. The amplification was carried out in a PCR 2720 Thermal Cycler (Applied Biosystems, USA). Further, the obtained amplicon was stained with Safe View (NBS Biologicals, Huntingdon, England) and separated on 1% agarose gel carried out in 0.5x TBE buffer (45 mmol/L Trisborate and 1 mmol/L EDTA) for 50 min at 100 V, using a VWR Mini Еlectrophoresis system (VWR, Germany) and MiniBis Pro (DNR Bio-Imaging Systems, Israel) for gel visualization. The PCR product was cut out from the gel and purified with Clean-Easy™ Agarose Purification Kit (Canvax Biotech, S.L., Spain).

Sequencing of the PCR product was performed by Microsynth Seqlab (Göttingen, Germany). The resulting sequence was analyzed using BLAST algorithm [46] and compared with the nucleotide sequences in the GenBank database [47]. The new sequence was deposited in the GenBank database and the accession number was assigned.

### Phylogenetic analysis

The phylogenetic analysis was conducted using the closest matched sequences from the GenBank database [47], sequences derived from open database from Hsieh et al. [48], Hsieh et al. [12], Thomas et al. [49], Peršoh et al. [39], Fournier et al. [2], U’Ren et al. [50], Pan et al. [9], Hashemi et al. [51], Rönsch et al. [52], Cañón et al. [4], Vega et al. [53], Jaklitsch et al. [54], Chen et al. [55], Del Olmo-Ruiz et al. [56] and unpublished sequences from the GenBank database [47] (Table 1). *Hypoxylon fragiforme* JN979420 [12] was used as an outgroup reference.

**Table 1:** Species, origin and GenBank accession numbers of sequences used in this study.

Species	Substrate / Origin	GenBank №	Reference
*Xylaria apoda*	Bark / China Taiwan	GU322437	Hsieh et al. [12]
*Xylaria cinerea*	‐‐‐ / France	FN689799	Fournier et al. [2]
*Xylaria coccophora*	Marine algae / Brazil	MG747437	Honorio et al. [47]
*Xylaria crozonensis*	Bark / France	GU324748	Hsieh et al. [12]
*Xylaria cubensis*	Plants / USA	JQ760658	U’Ren et al. [50]
*Xylaria curta*	Plants / China	GU322444	Chen et al. [55]
*Xylaria grammica*	Wood / China Taiwan	GU300097	Hsieh et al. [12]
*Xylaria hedyosmicola*	Fallen leaves / China Hainan	MZ227023	Pan et al. [9]
*Xylaria hypoxylon*	Wood / Belgium	GU300096	Hsieh et al. [12]
*Xylaria hypoxylon*	Leaf debris / Sweden	AM993146	Peršoh et al. [39]
*Xylaria karsticola*	Tree / Ecuador	MF770879	Thomas et al. [47]
*Xylaria karsticola*	‐‐‐ / France	FN689802	Fournier et al. [2]
*Xylaria karsticola*	‐‐‐ / France	FN689803	Fournier et al. [2]
*Xylaria lindericola*	Fallen leaves / China Hainan	MZ005636	Pan et al. [9]
*Xylaria longissima*	Wood / Iran	KP218906	Hashemi et al. [51]
*Xylaria muscula*	Dead branch / French West	GU300087	Hsieh et al. [12]
*Xylaria oxyacanthae*	Fruits / Germany	HQ414587	Rönsch et al. [52]
*Xylaria polymorpha*	Wood / USA	GU322460	Hsieh et al. [12]
*Xylaria polymorpha*	Stump / Germany	FM164944	Peršoh et al. [39]
*Xylaria polymorpha*	‐‐‐ / France	FN689809	Fournier et al. [2]
*Xylaria schweinitzii*	Tree / Ecuador	KP133455	Thomas et al. [49]
*Xylaria vasconica*	‐‐‐ / France	FN689804	Fournier et al. [2]
*Xylaria* sp.	Lichens / Brazil	KY962975	Cañón et al. [4]
*Xylaria* sp.	Yew / Iran	KF573972	Jam Ashkezari et al. [47]
*Xylariaceae* sp.	Leaf / Hawaii	EU009986	Vega et al. [53]
*Xylariales* sp.	Plants / Central and South America	KU747827	Del Olmo-Ruiz et al. [56]
*Hypoxylon fragiforme*	Bark / France	JN979420	Hsieh et al. [12]

The phylogenetic tree was obtained by means of the Unweighted Pair Group Method using the Arithmetic Average (UPGMA) clustering algorithm [57] and CLC Genomics Workbench 20.0 [58].

## Results

The fungus was isolated as sterile mycelium without any reproductive structures and was *in vitro* cultivated on MCM agar. The conventional methods for identification were not applicable and the cultural and morphological features (colony color, surface morphology, stroma production, conidial and conidiophore morphology) were observed on daily basis. The molecular identification was essential for the proper identification.

### Phylogenetic analysis

The phylogenetic analysis of the *Xylaria* isolate was performed using ITS1-5.8S-ITS2 region sequence data. The resulting sequence was analyzed using BLAST algorithm and compared with the nucleotide sequences in the GenBank database [47]. The strain was identified as *Xylaria karsticola* with 97.57% of confidence and the new sequence was deposited in the GenBank database under the accession number MW996752.

**Fig 2:**
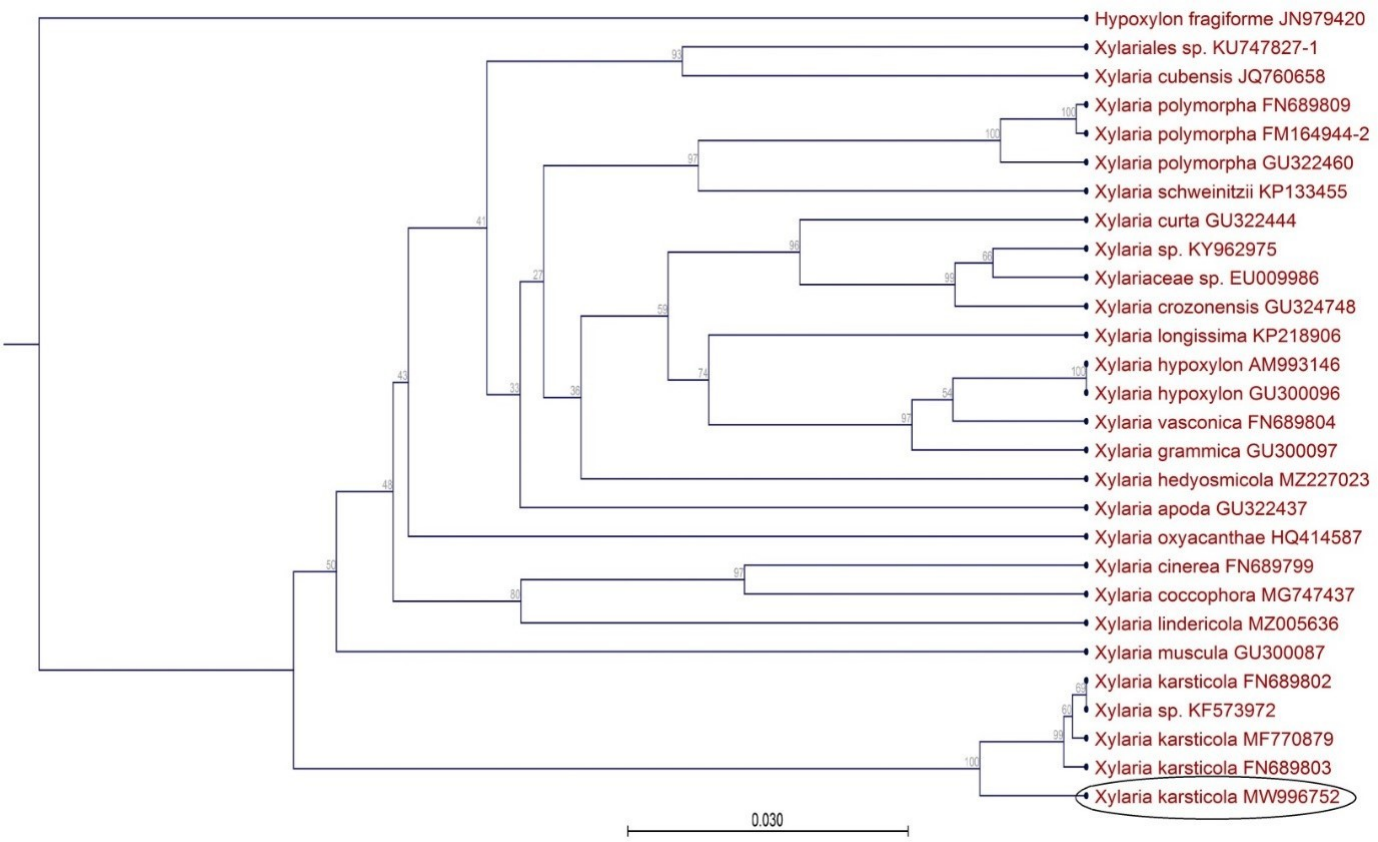
Phylogenetic relationship between *X*. *karsticola* MW996752 and other *Xylaria* spp., based on ITS1-5.8S-ITS2 sequence data.

The ITS1-5.8S-ITS2 rDNA gene sequence of the fungal isolate was compared with the total number of 27 sequences, including the closest matched sequences by a BLAST search and sequences derived from open database [2, 4, 9, 12, 39, 47–56]. The phylogenetic analysis clearly demonstrated the appearance of the new fungal isolate in a clade together with all other *X*. *karsticola* isolates (Fig 2).

However, the DNA sequence of *X*. *karsticola* MW996752 was rather distantly related to the other *X*. *karsticola* sequence data. The result was completely supported by the bootstrap analysis (100%) and indicated the different origin of the examined newly isolated *X*. *karsticola*. All *X*. *karsticola* sequences, included in the phylogenetic analysis, clustered at a long branch, confirming a quite distant relationship to the other *Xylaria* species used in this study. Relationship among some of the other *Xylaria* species could not be determined as the bootstrap support values of the respective branches were below 70%.

### Cultural and morphological characterization of fungal isolate

*Xylaria karsticola* J. Fourn. & M. Stadler, Mycological Progress 2011 Vol.10 No.1 pp.33-52 ref.39

NBIMCC № 9097.

GenBank № MW996752

**Fig 3:**
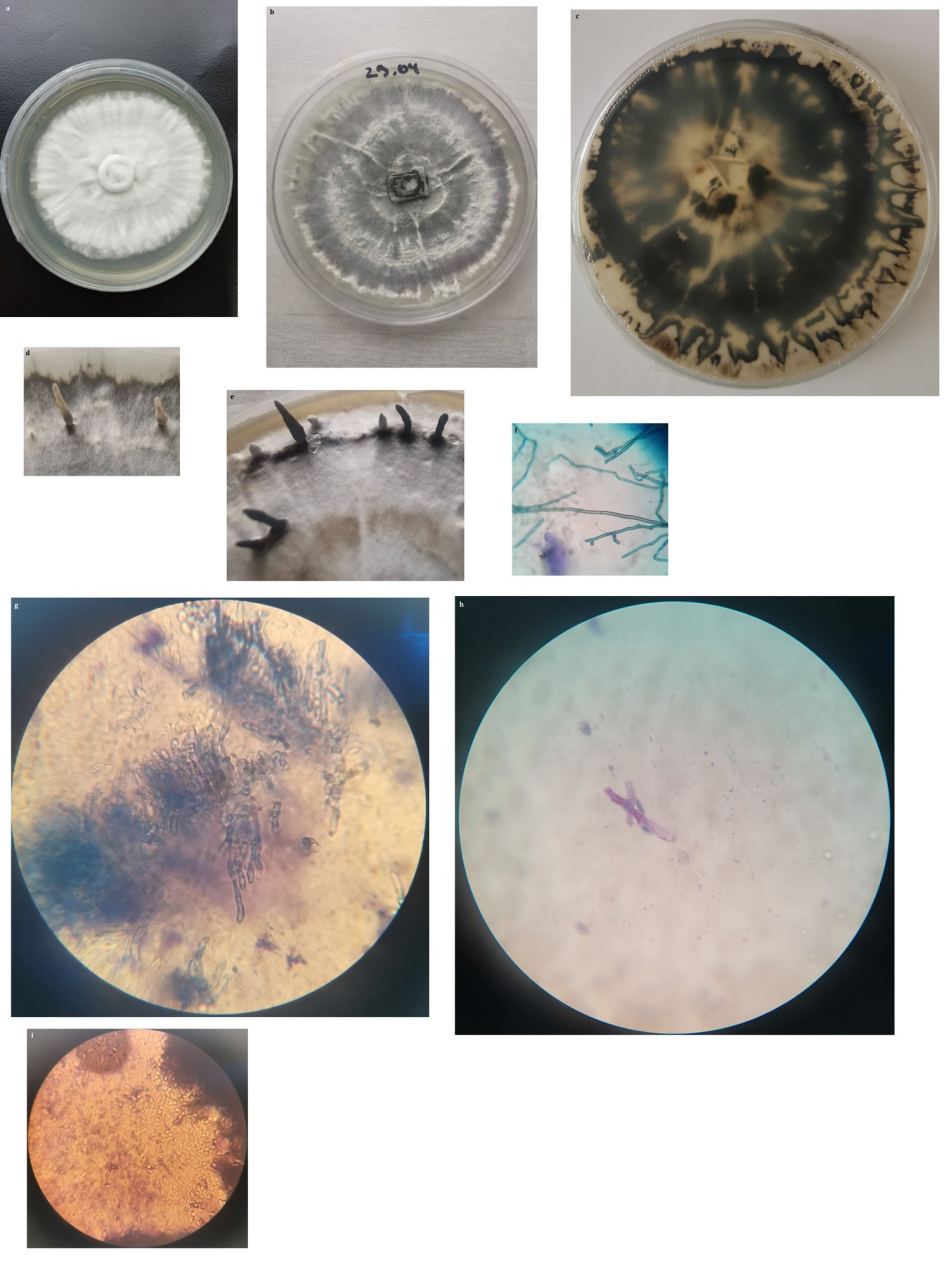
Colony of fungal isolate. (a,b), stromata formation (c,d,e) and microscopy feature of: mycelium (f), conidiogenous cells (g,h) and conidiospores (i).

Culture: The isolate demonstrated relatively fast radial growth rate with a peripheral fan-shaped extension spreading toward the edge of the petri dish (90 mm) for about 20 days, but not in all cases the culture reached the edge of the dish. At the early stage of cultivation, the colonies were white colored with cottony-like mycelium, zonate, with greyish and white concentric zones and diffuse margins. With aging dark grey to black color appeared (Fig 3A and 3B) and the bottom of the colonies became dark grey to near black colored (Fig 3C) The light microscopy observation of the mycelium showed that the hyphae are thin-walled, regularly septate with branches forming 90° angles (Fig 3F).

The first sign of a stromata structure formation became noticeable after 25 to 30 days of incubation.

Stromata: They arise from the fan-shaped region of the colony and become up to 10 mm high (limited by cover of dishes) and 2–3 mm thick (Fig 3C and 3D). The stromata are cylindrical, mostly monopodial, sometimes branch near to base. Initially they are greyish-white, covered by a conidial layer and with the time the color of the stromata changes to black or black with white apices (Fig 3E).

Stromata is an important diagnostically morphological structure for most of the genera belonging to *Xylariales* and we hypothesized that the fungal isolate belongs to genus *Xylaria*.

Conidiospores: Stromata never reach maturity and only anamorph is observed as cream-colored conidiogenous cells (Fig 3G and 3H), and covered by a white powdery mass of conidia over stromata apices. The elongate elliptical conidia, produced holoblastically, 6.5–10 × 4.0–5 μm could be seen on persistent conidiophores covering the stromata surface (Fig 3I).

Ascospores: The stromata formed during *in vitro* culture-cultivation was teleomorphic sterile one and fertile perithecium and ascospores were not observed even after long incubation time.

Known distribution: France

Commentary: The absence of fertile teleomorphic structure makes proper identification to species level difficult, therefore, molecular identification was conducted in addition to the morphological characterization of the unknown fungal isolate. Sexual morph was also not observed and collected from its natural habitat. The strain was deposited in The National Bank of Industrial Microorganisms and Cell Cultures of Bulgaria under accession number NBIMCC 9097.

## Discussion

This study presented the second report on *X*. *karsticola* from Europe, based on the isolate from Bulgaria. *X*. *karsticola* was described by Fournier et al. [2] from France. It is pertinent to mention here that *X*. *karsticola* has been isolated from the basidiocarp of edible basidiomycete mushroom *M*. *procera* for the first time.

Most members of the genus *Xylaria* are common as parasite, saprophyte or endophyte that frequently develop their fruiting bodies mainly on dead wood and different plant substrates [7, 9]. Wan-Rou et al. [28] reported about fifteen species of xylariaceous fungi isolated from the basidiomata of more than 100 *Scytinopogon* species. Some of these xylariaceous fungi were also found to be endophyte in nearby plants. According to this research xylariaceous fungi could establish coexistence with *Scytinopogon* sp. and these were referred to as fingicolous fungi. These fungi are large and diverse group able to associate with other representatives of the fungal kingdom [24, 28, 59]. They could be symbionts, saprotroph, mycoparasites and even neutrals [24]. The scientific interest to this group of fungi is focused on interactions between fungicolous fungi and their fungal host [60, 61] as well as their metabolite profile [62, 63]. To date only one scientific report dealing with the associations between basidiomycetous and ascomycetous fungi has been published [28]. To understand the details of interaction between *X*. *karsicola* isolate and its host *M*. *procera*, more hosts specimens growing in different ecosystems are required to get statistically significant results as well as further investigations confirming co-occurrence patterns. Further phylogenetic survey using robust multi-locus datasets, are needed to accept or reject the hypothetical coexistence of *X*. *karsticola* with *M*. *procera*.

According the findings of Fournier at al. [2] the species of *X*. *karsticola* favors in karstic areas where the soil is stony and the stromata develops in narrow spaces between wood remnants, stones and soil. The peculiar ecology of *X*. *karsticola* seems to be an adaptation to the relatively dry environment encountered in woodlands on calcareous, karstic soil. Further natural habitat observations are necessary in order to confirm the distribution of *X*. *karsticola*, examined in this study.

The cultural characteristics and morphology are a reliable way for identification of xylariaceous fungi, but the limited taxonomic resolution of the asexual state (only morphological characters) often could be a reason for inaccurate identification [13, 17, 64].

In the present study, the isolated strain was preliminary determined as xylariaceous morphotype at the intragenus level in culture, based on colony growth rate, color, and stromatic structure formation that was confirmed by conidiophores and the conidia observed. According to Petrini and Petrini [13], colony growth rate, color, and stromata structure are more stable diagnostic characteristics for xylariales than conidiophores morphology and shapes and sizes the conidia because they don’t produce conidiogenous cells and conidia at any conditions.

The cultural characteristics and morphology of the isolated strain were compared to *X*. *karsticola (*JF 08171) isolated in France [2]. Our strain roughly resembles *X*. *karsticola* JF 08171 in the moderately rapid growth rate of the culture. There are differences between the two strains in the color of the colonies and reverse. Our strain is culturally and morphologically different also from *Xylaria vasconica* and *Xyldria hypoxylon* [2, 39].

The strain we observed produces stromata and anamorph structures after not so long cultivation period on MCM (4 weeks), most often arising from the fan-shaped region of the colony. Initially, the stroma is covered by white to greyish colored powdery mass consisting of conidiogenous cells and elongate elliptical conidia. The strain JF 08171 needs much longer incubation time before the initiation of stromata formation and the stroma they observed were restricted only to the centre of the colonies at the beginning and with time covers entire surface of the colonies. No conidiogenous cells were observed by the authors [2].

However, we noticed that when the strain is cultivated in medium with lignin and cellulose, the time for stromata and anamorph production is shorter and starts after 15 days of incubation. The similar findings were reported for *X*. *karsticola* JF 08171 by Fournier at al. [2]. These findings also supported the statements that the unfavorable conditions provoke sexual stage as well as conidiogenesis [65].

The strain we studied remains sterile in culture and we did nоt observe teleomorphic features–ascocarp and mature ascospores. The lack of mature stromata found in natural habitat and sexual cycle complicate determination with certainty the species-level identity of the isolate basing only on cultural and morphological characteristics and anamorphic feature, such as conidiogenous cells and conidia.

Molecular techniques have become the most powerful and essential tools in identification and phylogenetic survey of fungi, including *Xylaria* species [55]. Among all DNA markers, the ITS region is most commonly used for species delimitation. In the present study, a phylogenetic analysis of the novel, sporadic isolated *X*. *karsticola* was conducted, including 26 *Xylaria* isolates from different species. Our literature survey showed that there was only one previous published study, associated with *X*. *karsticola* in Europe [2, 3]. Considering the data generated by the phylogenetic analysis of ITS regions of rDNA, the examined *Xylaria* isolate was grouped within the same clade with other *X*. *karsticola* isolates. The DNA sequence of *X*. *karsticola* MW996752 was rather distantly related to the other *X*. *karsticola* sequence data, demonstrating the phylogenetic difference of the examined fungal isolate. A probable reason might be the lack of available DNA sequences from the GenBank database, corresponding to *X*. *karsticola*. The BLAST search shown only 97.57% of confidence with the closest matched sequence *X*. *karsticola* MF770879 [47], which differed with 4 bp (the branch length differed with 0.011). The relatively low percent of identity (97.57%) with other *X*. *karsticola* sequence data might be due to some interspecific interactions between the xylariaceous fungi and the host organism (*M*. *procera*). The presented phylogenetic analysis was in agreement with Fournier et al. [2], demonstrated that all *X*. *karsticola* sequences, included in the phylogenetic analysis, clustered at a long branch, confirming a quite distant relationship to the other *Xylaria* species used in this study.

Putatively novel *X*. *karsticola* NBIMCC 9097 isolated from fruiting body of *M*. *procera* was clustered with all other *X*. *karsticola* isolates, although it appeared to lack closely related, described species. According to U’Ren et al. [50], xylariaceous isolates identified as previously described species may in fact represent novel species, but inferences are limited by the potential for previously known *Xylariaceae* to be absent from public databases.

Additional studies are necessary to elucidate the affiliation of *X*. *karsticola* NBIMCC 9097 to the same species. With a relative lack of species-specific DNA barcodes and phylogenetic markers compared to many other ascomycete groups, field sampling is required to find putative stromata with teleomorphic structure of this species in order to provide information about the morphology of sexual structure and to confirm the identification.

Furthermore, this survey might be of a great significance for exploring the geographic distribution of the novel *X*. *karsticola*. The elucidation of the interaction between the studied in this work *X*. *karsticola* and the mushroom *M*. *procera* could enrich the knowledge about the xylarioceus fungi and their mycohost. Based on the *in vitro* cultivation this *X*. *karsticola* NBIMCC 9097 presents an interesting source for future screening of natural secondary metabolites with therapeutic properties.

## Supporting information

S1 Fig Certificate of deposition. (TIF)

S2 Fig ITS4 ampl. Numbers 1.1. and 1.2. mark the *X*. *karsticola* NBIMCC 9097 IST amplification.
(TIF)

S1 File ITS4 sequence of *X*. *karsticola* NBIMCC 9097. (DOCX)

S1 Raw images (PDF)
