# Supplementary figures and images for: Molecular and morphological characterization of Xylaria karsticola (Ascomycota) isolated from the fruiting body of Macrolepiota procera (Basidiomycota) from Bulgaria
Source: PLoS One. 2023 Jun 29;18(6):e0287679. doi: 10.1371/journal.pone.0287679 (PMC10309620; doi:10.1371/journal.pone.0287679)

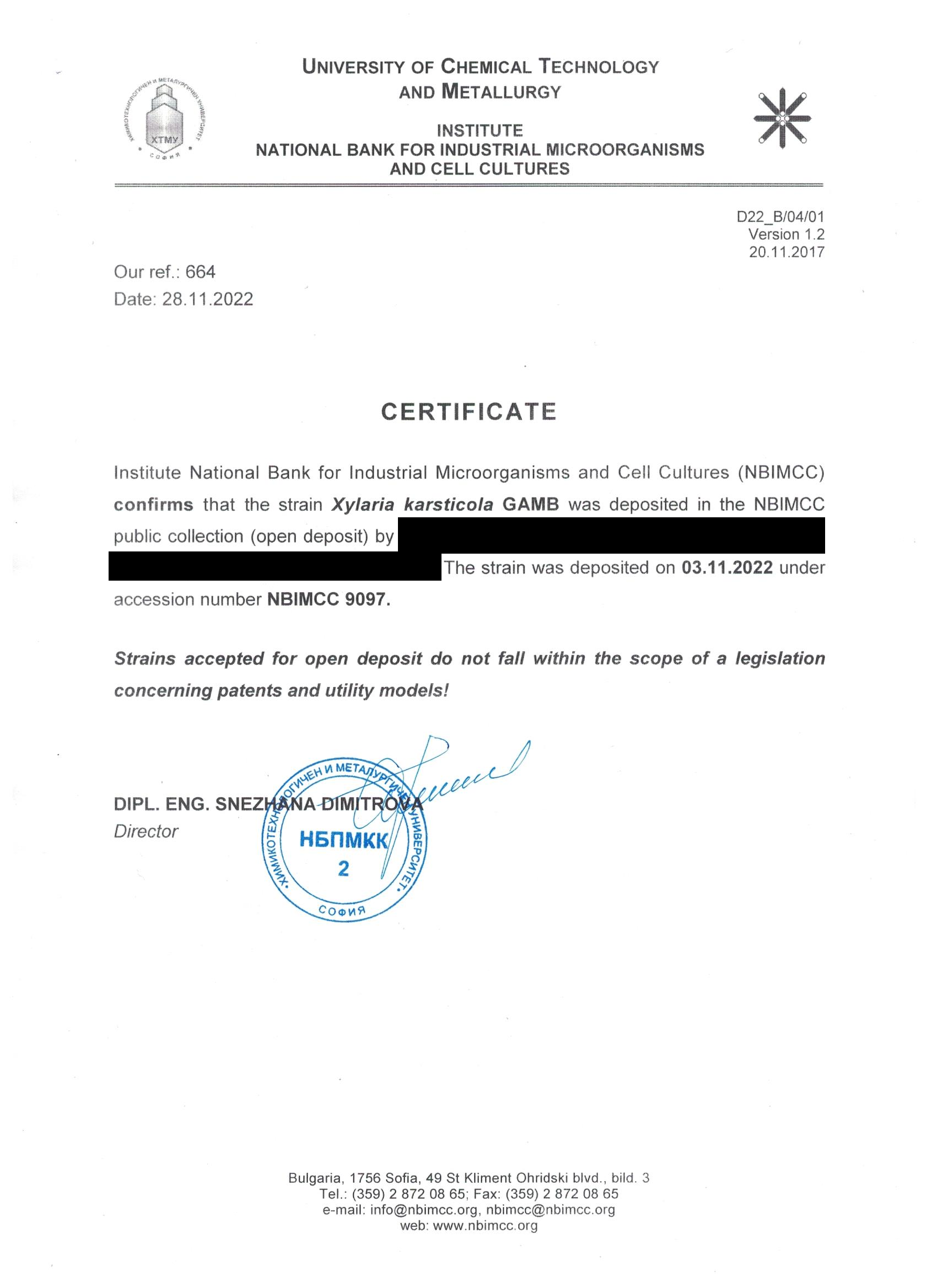

Supplement: S1 Fig — (TIF) [file pone.0287679.s001.tif]

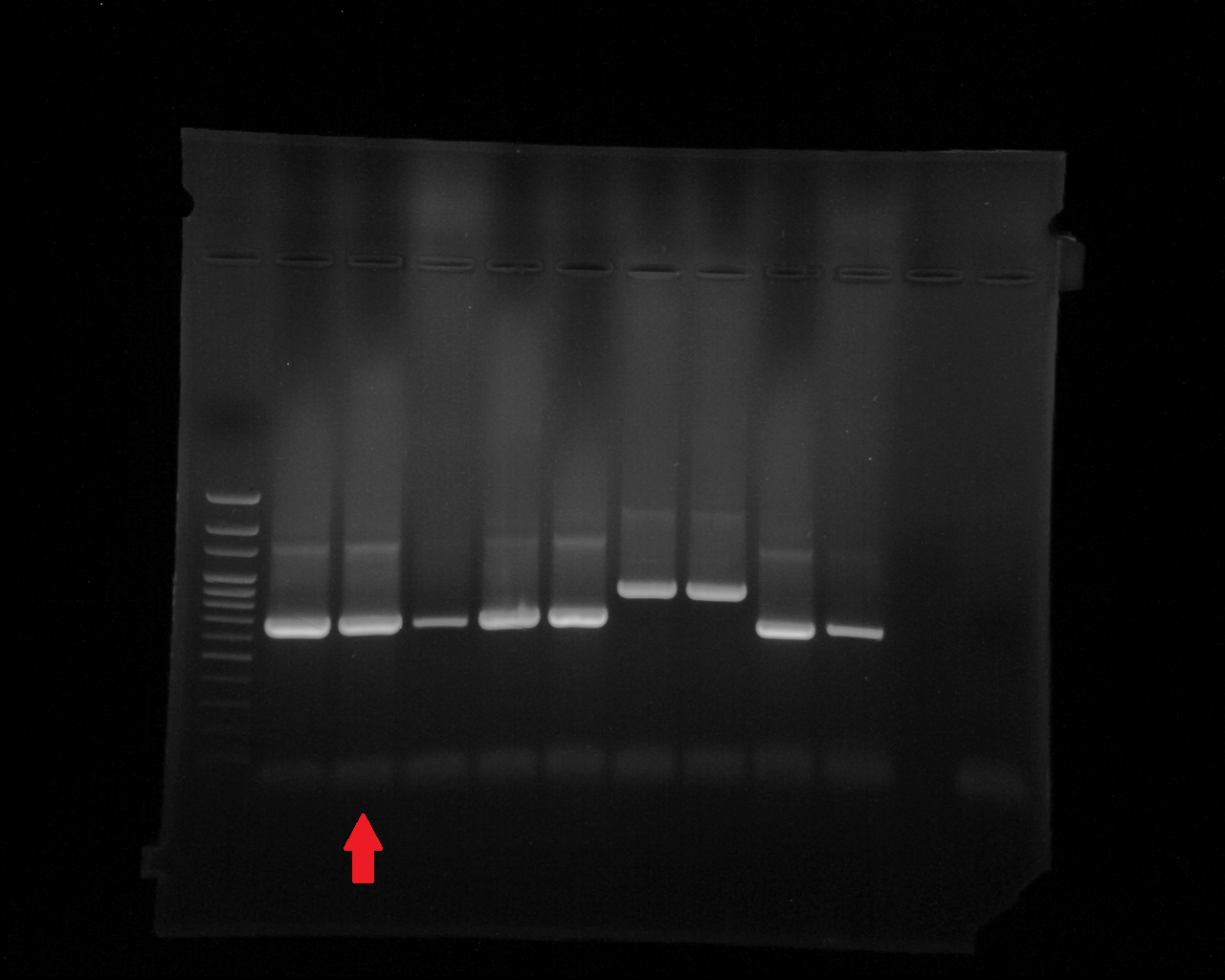

Supplement: S2 Fig — Numbers 1.1. and 1.2. mark the X. karsticola NBIMCC 9097 IST amplification. (TIF) [file pone.0287679.s002.tif]

M 1.1 1.2 X X X X X X X X X

3000 bp —  
2000 bp —  
1500 bp —  
900 bp —  
700 bp —  
1000 bp —  
800 bp —  
600 bp —  
500 bp —  
400 bp —  
300 bp —  
200 bp —  
100 bp —

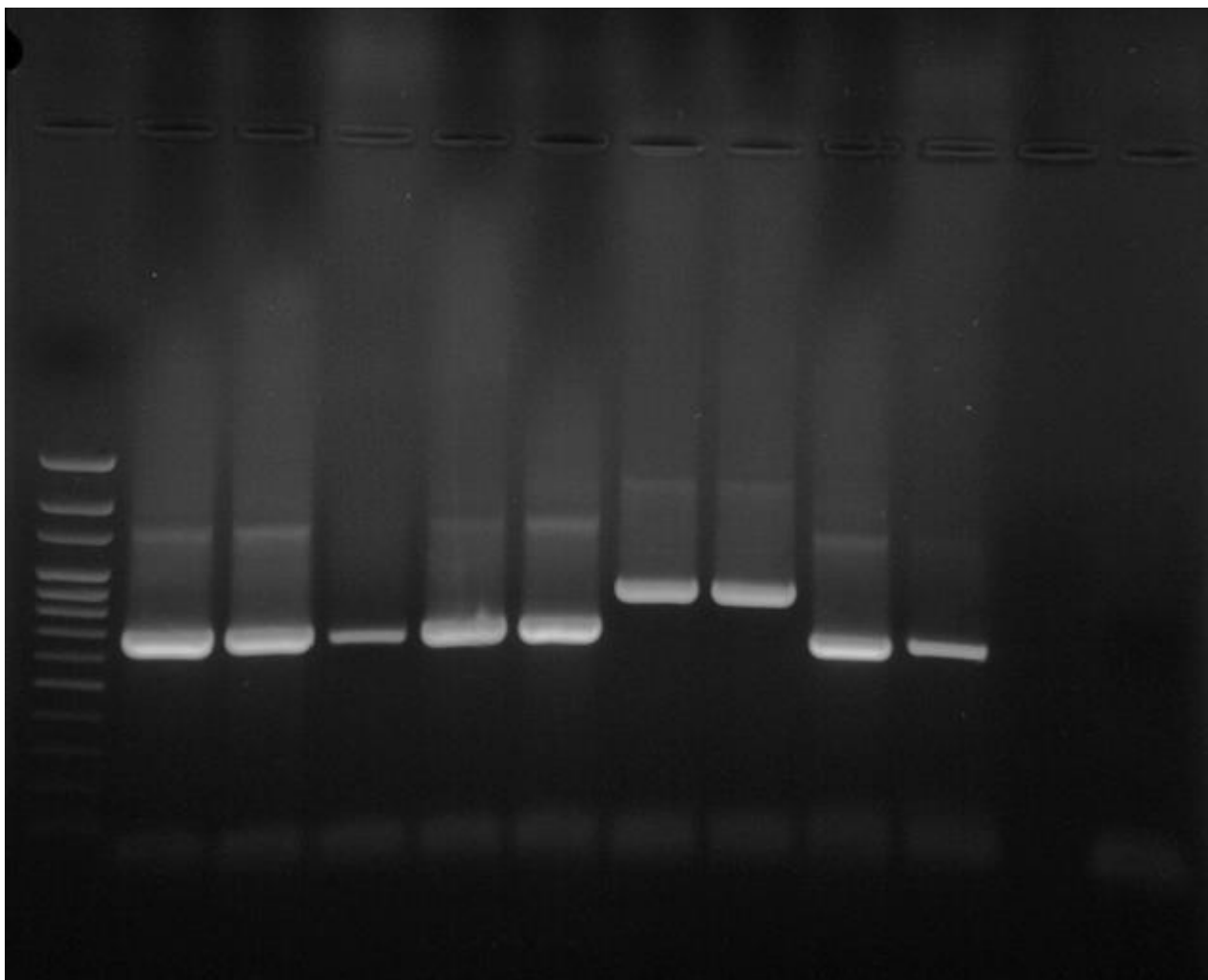

Supplement: S1 Raw images — (PDF) [file pone.0287679.s004.pdf]
