# Supplementary material for: Molecular and morphological characterization of Xylaria karsticola (Ascomycota) isolated from the fruiting body of Macrolepiota procera (Basidiomycota) from Bulgaria
Source: PLoS One. 2023 Jun 29;18(6):e0287679. doi: 10.1371/journal.pone.0287679 (PMC10309620; doi:10.1371/journal.pone.0287679)
Supplement: S1 File — (DOCX) [file pone.0287679.s003.docx]

>1_ITS4_01

TCCGAGGTCACCTTTAAAAAGTAGGGGGTTTTACGGCAGGAGATCGGGGCAGCTCTAGGCGAGATTAAAAATAATTACTACATCTAGAGCGTGAACCGATTCCGCCACTCAATTTAGGGAGCTACAGCTTTTACTGTAGGCCCCCAACGCTAAGCAACAGAGGCTTAAGGGTTGAAATGACGCTCGAACAGGCATGCCCACTAGAATACTAATGGGCGCAATGTGCGTTCAAAGATTCGATGATTCACTGAATTCTGCAATTCACATTACTTATCGCATTTCGCTGCGTTCTTCATCGATGCCAGAACCAAGAGATCCGTTGTTGAAAGTTTTAATAATTTCTTATTTAGGTTCAGAATAACATAATAAACAGAATTTAATAGGCCACCGGCAGGCAGGGCTATACCGTGTCGTCACACGGTATAACACAACCTGCCGAGGCAACAACAGTGGTAAGTTCACATGGGTTAGGGAGTTTTATACTCTCTTTAATGATCCCTCCGCTGGTTCACCAACGGAGACCTTGTTACGAT
